# Prediction of arabica coffee production using artificial neural network and multiple linear regression techniques

**DOI:** 10.1038/s41598-022-18635-5

**Published:** 2022-08-25

**Authors:** Yotsaphat Kittichotsatsawat, Nakorn Tippayawong, Korrakot Yaibuathet Tippayawong

**Affiliations:** 1 Graduate Program in Industrial Engineering, Faculty of Engineering, Chiang Mai University, Chiang Mai, 50200 Thailand; 2 Excellence Centre in Logistics and Supply Chain Management, Chiang Mai University, Chiang Mai, 50200 Thailand; 3 Department of Mechanical Engineering, Faculty of Engineering, Chiang Mai University, Chiang Mai, 50200 Thailand; 4 Department of Industrial Engineering, Faculty of Engineering, Chiang Mai University, Chiang Mai, 50200 Thailand

**Keywords:** Engineering, Environmental social sciences, Sustainability, Computational science, Computer science

## Abstract

Crop yield and its prediction are crucial in agricultural production planning. This study investigates and predicts arabica coffee yield in order to match the market demand, using artificial neural networks (ANN) and multiple linear regression (MLR). Data of six variables, including areas, productivity zones, rainfalls, relative humidity, and minimum and maximum temperature, were collected for the recent 180 months between 2004 and 2018. The predicted yield of the cherry coffee crop continuously increases each year. From the dataset, it was found that the prediction accuracy of the R^2^ and RMSE from ANN was 0.9524 and 0.0784 tons, respectively. The ANN model showed potential in determining the cherry coffee yields.

## Introduction

Coffee is one of the most popular global beverages, and Thailand is among the world’s top 25 coffee exporters, with over 30,000 tons per year^1^. It can generate enormous earnings for the country’s economy and farmers. It was found that high-standard coffee can get an asking price a few times more than standard products^2,3^. However, coffee products have been encountering several problems, including unqualified beans, climate change and environmental effects, a decrease in the nutrition level in soils, pests and insects, a lack of farming knowledge management, as well as the unavailability of raw materials^4–7^. While the main focus should be on the quality of the products to satisfy customer requirements, the lack of management might be the leading cause of these problems and might affect the efficiency of the accuracy rate. This could also subsequently lead to the failure of marketing prices in the future^8,9^. At present, the world's coffee consumption has grown by an average of over 2% a year in the past decade^10^. The Thai coffee industry has shown similar growth between 2016 to 2020. Thailand's average demand for coffee beans is almost 79,000 tons per year^11^. Accurate prediction of coffee will improve farmers’ or entrepreneurs’ ability to plan their annual coffee production and reduce the risk of inconsistent demand, supply, and lack of trade opportunities.

**Figure 1:**
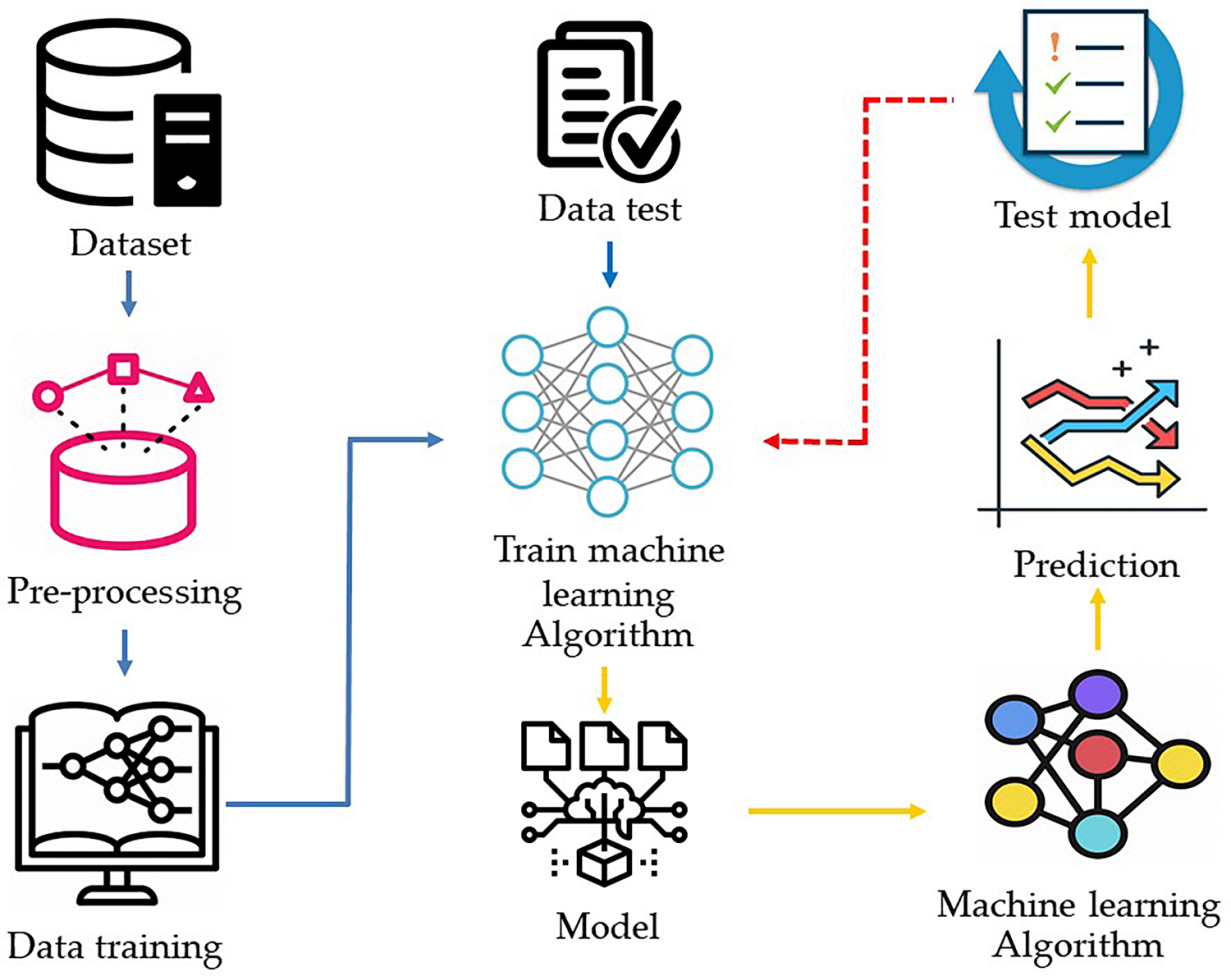
Machine learning predictive models.

Arabica coffee (*Coffea arabica* L*.*), one of the main coffee species, has been considered a suitable plant to promote as an opium substitute to stop shifting cultivation^12,13^. However, Arabica is also one of the most popular species of coffee that customers look for because it features a particular characteristic flavor that differs from other types of coffee^14^. For this reason, farmers and entrepreneurs are likely to concentrate on Arabica coffee to meet the demand from customers. Hence, it is necessary to predict each year's yields in order to manage the insufficient supply and the overwhelming demands of customers so as to reduce the loss of trade opportunities. Forecasting applications have recently been used to predict demand, supply management, and resources to reduce mistakes in terms of overproduction and operating costs^15,16^. Prediction in agriculture is mainly made in terms of rainfall estimates, area, production or productivity, and price^17,18^. Thus, these factors should be used to predict the productivity and yield of agricultural products, possibly with a machine learning model, as shown in Fig. 1.

Machine learning (ML) is carried out to learn and make decisions without setting rules and conditions^19^ because it can learn through reading human-provided information and processing data. This data information can be applied to make the desired decisions^20^. The decision-making of ML has various formats, for example, predicting and classifying a new database^21^, identifying and clustering with different data^22,23^, detecting incorrect data information^24^, and introducing new data that are expected to interest humans by learning from previous interests^25,26^. In our problem, the multilayer perceptron was adopted for prediction. Neurons in the network are generated in a layer. Then, the output of one layer supports the input into the next layer and conceivably others. The model parameter of this study can be optimized by using the feed-forward backpropagation training algorithm. The ML model has great potential for application to coffee production prediction. Therefore, this research uses artificial neural networks (ANN) in coffee cherry prediction because of its ability to learn the patterns of complex systems. Moreover, it can predict future productivity from the current database more effectively than the traditional statistical method.

ML is critical to studying programming and computer design, such as robotic systems, visual systems, or computer vision^27–29^. A computer algorithm generates the machine learning program, and this program is then able to calculate and evaluate the condition, and the software will generate the decision-making. Then, the data is transferred to the dataset in order to generate the model. After that, the completed program is applied to determine work usability^30^. ML can be categorized into two groups; (i) supervised learning, which comprises regression and classification groups, and (ii) unsupervised learning, which comprises clustering and non-clustering groups^31^. ML uses a variety of algorithms, for example, linear regression, logistics regression, support vector machines, decision trees, random forest, boosting, convolutional neural network, gradient-boosted trees, and ANN^32–34^. In this work, we are interested in ANN.

The ANN model is now considered a vital data-modeling tool^35^. It is a calculation model that activates the functional structures of biological neural networks. A neuron is one part of the process running unit that acquires inputs and consequently operates these inputs to achieve outputs. The construction of the ANN includes many architectures, consisting of single-layer perceptrons, multilayer perceptrons, recurrent ANN, and self-organization mapping. Each of these is appropriate for different problems^36^.

The significant benefit of the ANN technique is the capability of modeling multiple outputs concurrently^37^. Improvement of the ANN model requires data generation for training or testing, selecting the optimal configuration, and validating the model on an independent data set^38^. The optimal configuration was found to be 1 and 2 hidden layers, 2–10 neutrons, and 1000–10,000 learning runs^39^. ANN is connected via a structure of single-layer perceptrons and consists of input, output, and hidden layers, which reconstruct the input through the output layer. ANN analysis was used to analyze the input and output data using the MATLAB Neural Network Toolbox. It includes input, hidden, and output layers, which are the central elements of each ANN structure^40^. The input layer will obtain the input data and normalize from datasets through neurons. Then, all of the data will be transmitted to the hidden layer^41^. Every neuron of the subsequent layer(s) will calculate the output data, transmitting a linear combination of all the neurons in the previous layers^42^.

ANN is a technique that uses data mining. A mathematical model is used to connect the information processing and computation and simulate the function of neural networks^43^. Neurons in computer systems include input and output, with neuron weights that can indicate the input and output weights. Then, the threshold value is the decision for transferring the output data through the neurons. After the collaboration work is finished, the results are shown in numerical calculations format^36^.

Previously, ANN and MLR have been employed in many various fields, such as irrigation groundwater quality parameters^44^, forecasting PM levels^45^, predicting SO_2_ concentration^46^, slope stability^47^, intrinsic solubility of generic drugs^48^, electrical conductivity^49^, mosquito abundances in urban areas^50^, estimating monthly rainfall distribution in Thailand^51^, prediction of human skin permeability^52^, etc. Presently, ANNs are used in medicine and health care^53,54^, medical image denoising^55^, face detection and recognition^56^, road and traffic signaling^57^, road sign detection and recognition^58,59^, and in modeling conventional and solar earth^60^. ANN and MLR are also currently used in agriculture, for instance, predicting organic potato yield^37^ and the yield in the hulless barley^61^.

It can also be used in the agricultural field to detect and classify plant diseases, crops, and pests, especially in coffee bean species and coffee cherry^62^. Ahmad et al.^63^ applied ANN to develop an automatic coffee sorting system based on image processing. Moreover, ANN was used to evaluate the degree of coffee roasting, predicting the sensory quality of a roasted coffee-flavored sterilized drink and recognizing the degree of coffee roasting^64^. ANN could be applied to predict the amount of agricultural production by using previous datasets in order to obtain correct information through a machine learning-based algorithm for annual coffee production^65^. In addition, ANN can predict not only agricultural products but also enter many types of data and extract knowledge outside of the primary dataset. Torkashvand et al.^66^ applied the ANN and MLR model to evaluate and improve the accuracy rate in predicting kiwifruit firmness. Dhyani^67^ used the ANN and MLR model to measure the rainfall to anticipate the crop in the following year. Furthermore, Etminan et al.^68^ utilized an ANN model to identify the best drought tolerance indices in agronomy and plant breeding by providing this valuable model in other biological contexts.

According to the literature, ANN is one of the tools that is suitable for predicting agricultural products because it can simulate the physical behavior of a complex system from the input data. The advantages of ANN are as follows; (i) it can give a more accurate prediction than general methods, (ii) the model can still be used even if there are errors in the actual data, (iii) any conflicting results can still be implemented got either are continuous, (iv) the analysis of results is fast after the learning process^69^. On the other hand, there are disadvantages of ANN; (i) a long training time can be necessary in order to find the best weight to learn, (ii) it is not easy to choose suitable parameters (the best topology), (iii) the learned function can be difficult to understand^70^.

MLR is used to study the collaboration of the relationships of two or more variables that affect another value. The regression includes various linear regressions and logistic regression algorithms that control the learning model to predict the input data into the program^71^. Its algorithms are more complicated but are also used in agricultural production, for example, ordinary least squares regression, multivariate adaptive regression splines, multi-linear regression, and locally estimated scatterplot smoothing^72^.

**Figure 2:**
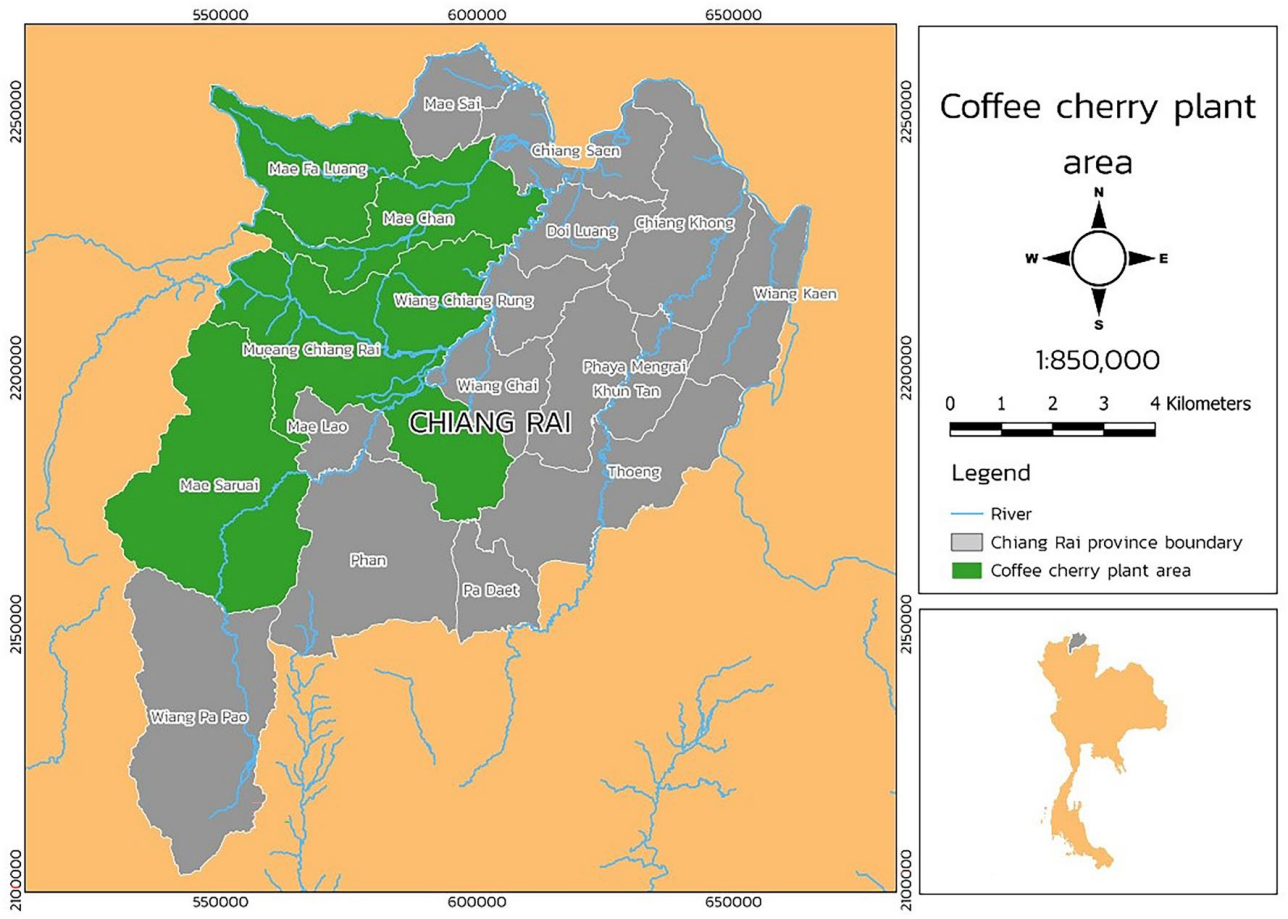
Coffee plantation area in Chiang Rai, Thailand. Figure has been generated by Mr. Yotsaphat Kittichotsatsawat, the first author of this manuscript using the Qgis program. Version 3.8.1. URL. https://www.qgis.org/en/site/index.html.

From the literature review, it was noticed that ANN and MLR are not yet utilized widely in agricultural production. Their applications are relatively novel in the study of agricultural production. To the authors' knowledge, only a few works have deployed ANN and MLR to predict agricultural crop yield. So far, no work has been reported on applying machine learning to coffee cultivation. This study investigates and analyzes the prediction of cherry coffee and quantity control during plantation before processing the roasted coffee beans in Chiang Rai in northern Thailand, as shown in Fig. 2.

## Results

### Descriptive statistics

In this study, we provided the predicted yield of arabica coffee for 15 years from 2004 to 2018. Coffee is seasonally produced and available 6 months a year. Off-season, there is no yield. The dataset used in the study consists of factors related to crop yield, including the area, productivity zone, monthly rainfall, monthly RH, and monthly temperature. The data source is from the northern part of Thailand, which has been collected for 180 months. A summary of the statistical characteristics of the dataset is presented in Table 1. It was shown that the productivity zone had a positive skewness distribution of 0.413, and the temperature had a negative skewness distribution of − 0.467. The other variables were slightly skewed. The following subsection shows the results of the modeling. Productivity, also known as crop yield, is the variable to be predicted using the MLR. To apply MLR modeling, the correlation between pairs of input variables was examined to decide which variables to include in the model. The results are shown in Table 2.

**Table 1:** Summary of crop yield statistics.

Variable	Maximum	Minimum	Mean	Median	SD	Skewness	Kurtosis
Area (hectare)	42,215	20,158	34,003	31,186.5	11,028.5	0.292	0.336
Productivity zone (hectare)	37,710	14,513	23,921	26,111.5	11,598.5	0.413	− 0.979
Monthly total rainfall (mm)	509.8	0	1830	254.9	254.9	− 0.055	− 0.739
Relative Humidity (%RH)	82.0	55.0	76.0	68.5	13.5	− 0.168	− 0.788
Temperature monthly (°C)	40.0	5.10	25.5	22.6	17.5	− 0.467	0.258
Productivity (tons)	4794.27	365.066	2505	2579.7	2214.6	− 0.243	− 1.106

**Table 2:** Correlation matrix of the input dataset.

Variable	Area	Productivity zone	Rainfall	RH	Tmax	Tmin
Area (hectare)	1.0000					
Productivity zone (hectare)	0.8119	1.0000				
Monthly total rainfall (mm)	0.0100	0.0677	1.0000			
Relative Humidity (%RH)	− 0.3366	− 0.1641	0.2527	1.0000		
Tmax monthly (°C)	0.6278	0.5140	− 0.1882	− 0.6542	1.0000	
Tmin monthly (°C)	0.3233	0.5962	0.1790	0.3080	0.3192	1.0000

In the MLR model, it is common to include the input variables with a high linear correlation with the response variable but to exclude ones that are highly correlated among themselves because of the multicollinearity problem. Table 2 shows that the area and productivity zone variables are highly positively correlated, while the variables Tmax and area and Tmax and RH are moderately positively and negatively correlated, respectively. These results might lead to a multicollinearity problem; however, in this study, we included all variables in the MLR, as the dataset is one hundred eighty observations and six variables. The results are shown in “Prediction results”.

### Prediction results

**Figure 3:**
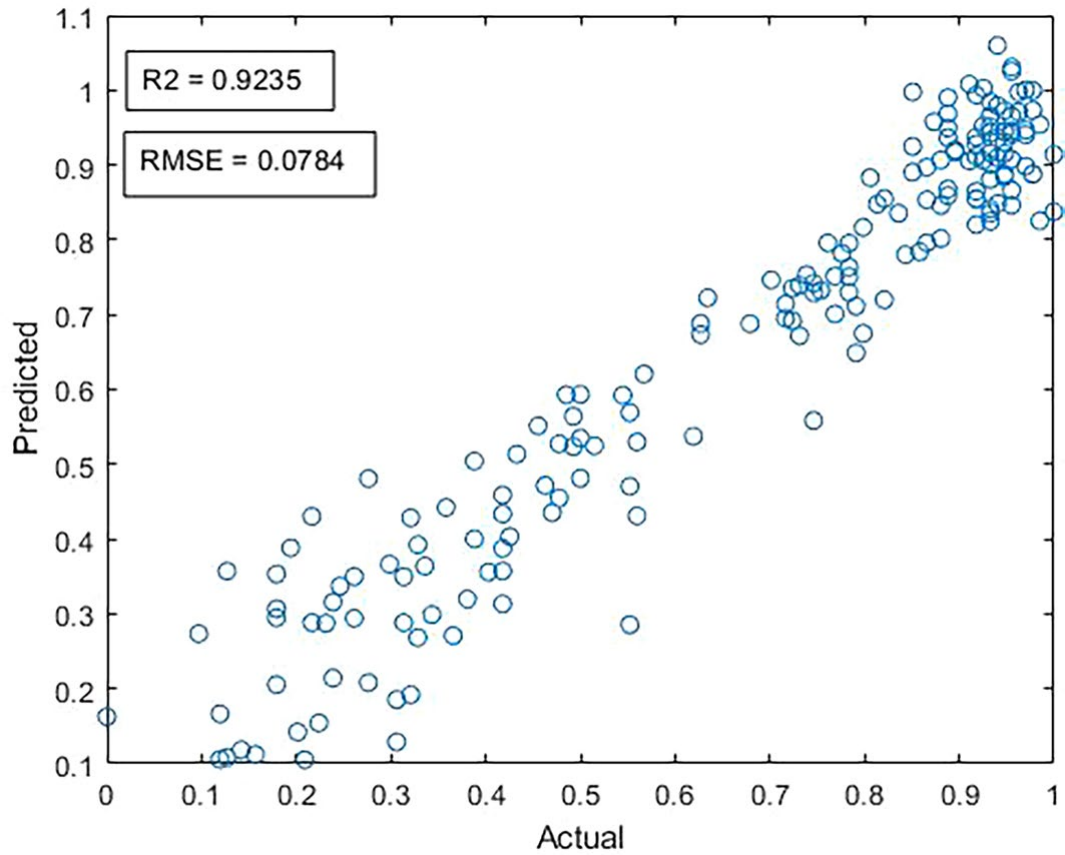
The R^2^ and RMSE (ton) of the scatter plot on the MLR model between the actual and predicted crop yield.

The MLR, in which the equation was derived from the full model of six variables, is used to predict crop yield. The MLR equation, R^2^, and the Durbin‒Watson test are shown in Table 3. From Table 3, the model that includes six input datasets yields a high value of R^2^, which indicates that the coffee features substantially explain the change in the crop yield (92.35%). Meanwhile, the Durbin‒Watson test is 1.56, which reveals a suitable autocorrelation for the predictive errors. After data analysis, it was found that the R^2^ is 0.9235, RMSE is 0.0784, and MSE is 0.0061 ton, which is suitable for the model (see Fig. 3).

**Table 3:** Results of MLR analysis to estimate crop yield of cherry coffee productivity.

R^2^	RMSE	MSE	Durbin–Watson	Equation (coefficient)	P-value
0.9235	0.0784	0.0061	1.56	Crop yield = 0.2850 + 0.0402 Area + 0.3128 Productivity Zone − 0.0486 Rainfall − 0.4040 RH − 0.6144 Tmax + 1.1065 Tmin	0.001

From Fig. 3, when the MLR model tested the input data and crop yield, it can be seen that the linear relationship was preserved between the variable input datasets and the crop yield. Thus, farmers and entrepreneurs could use this data set to predict cherry coffee productivity in the next period.

The ANN analysis was performed based on the previous 180 months’ input dataset. In all ANN analyses, the input and output had eight neurons and one layer. The number of neurons in the hidden layer was defined using trial and error to find the best ANN configuration for predicting the crop yield of cherry coffee. The ANN configuration determined to be optimal should minimize the MSE and RMSE and optimize R^2^. The ANN configuration from the network topology started from one and then consecutively increased.

**Figure 4:**
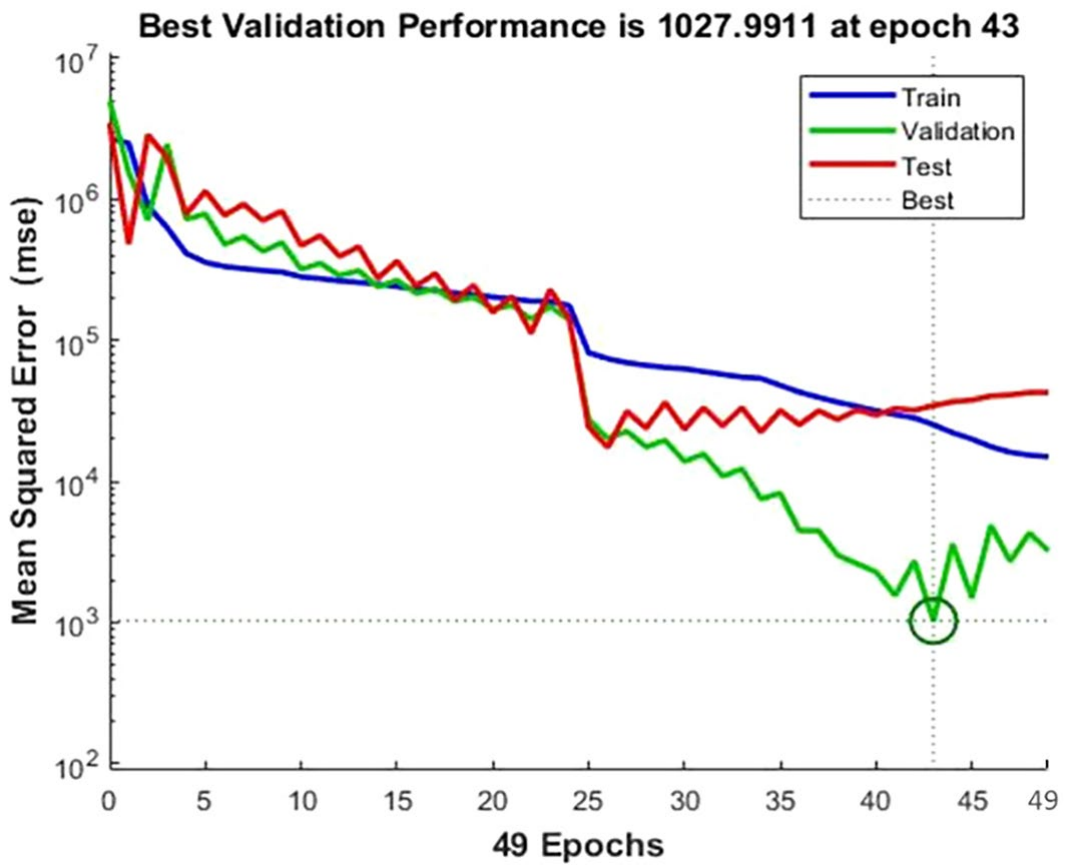
Mean squared error (MSE) of cherry coffee validation performance.

**Figure 5:**
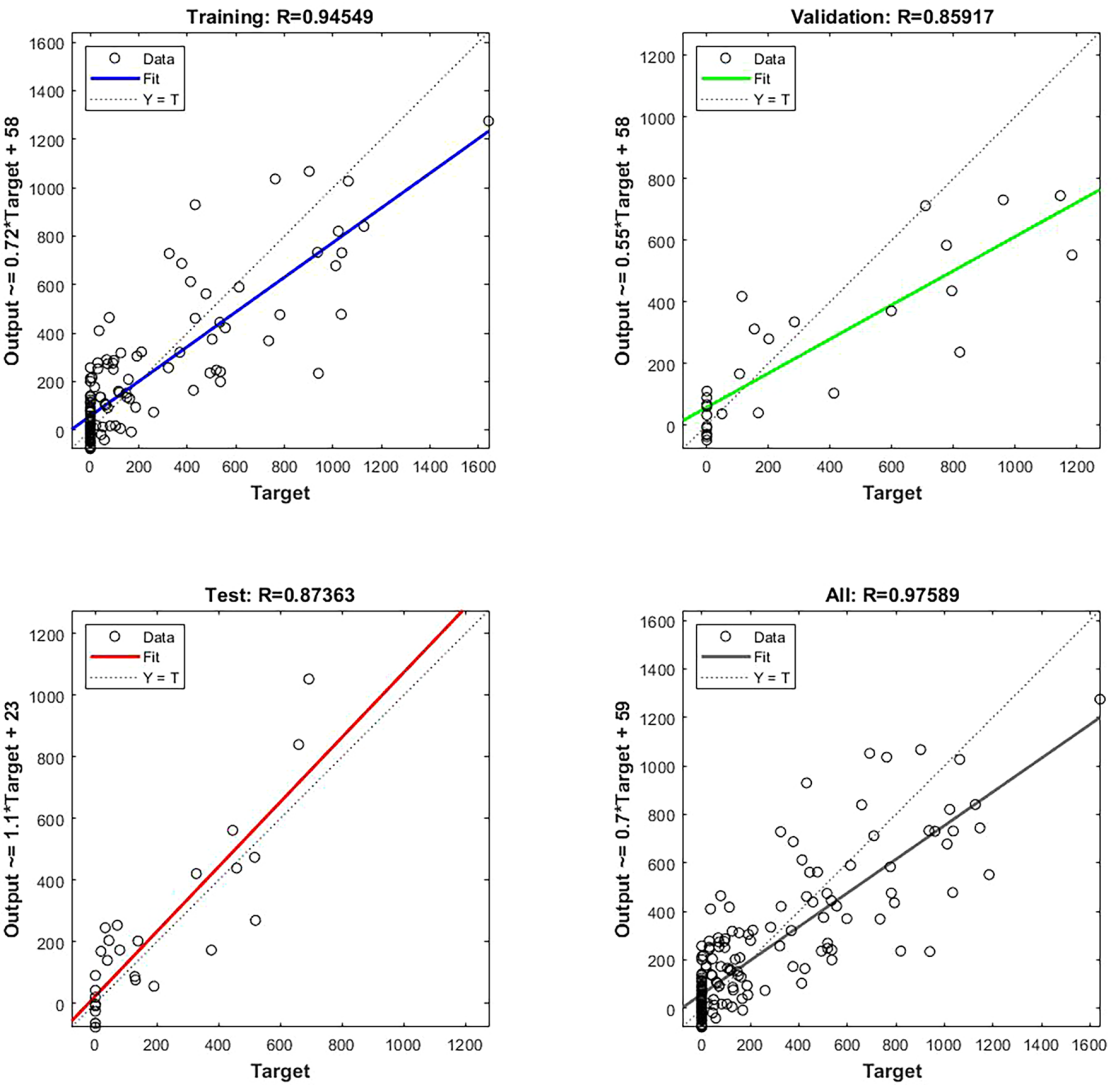
Prediction of the coffee cherry using ANN (MSE training and predicted values of cherry coffee by multilayer perceptron neural network versus data value, training and validation phases) (ton).

From Table 4, the network properties will set the treatments of the (i) type of network, (ii) input and target data, (iii) training and adaptation learning function, (iv) performance function, (v) layer number, (vi) number of neurons, and (vii) transfer function. Feed-forward backpropagation was used for setting the network type. The number of hidden layers is fixed as two, numbered H1 and H2, and the number of neurons will set the number of processing elements. Then, these data will be trained in order to obtain the best ANN performance. After training the ANN model, the best of the various ANN configurations is with the second hidden layer and the eighth processing element. Meanwhile, the MSE and R^2^ are 1027.99 and 0.9524 tons, respectively, as shown in Figs. 4 and 5.

**Table 4:** The R and MSE (ton) performances of various ANN configurations using once trained for the dataset.

Hidden layer (Hl) number	Processing elements (PEs)	MSE	R
1	1	34,115.10	0.777
1	2	316,012.50	0.757
1	3	287,720.50	0.757
1	4	9384.50	0.746
1	5	2102.34	0.910
1	6	241,512.50	0.684
1	7	23,690.64	0.922
1	8	55,592.50	0.776
1	9	409,160.00	0.859
1	10	84,228.49	0.888
2	1	72,170.25	0.857
2	2	9383.68	0.684
2	3	50,069.96	0.859
2	4	66,932.68	0.871
2	5	87,831.30	0.541
2	6	53,020.86	0.851
2	7	5150.58	0.921
2	8	1027.99	0.975
2	9	103,884.14	0.855
2	10	113,635.85	0.761

Figure 4 shows the MSE from the random data generated, which included the training, validation, and testing data. The learning rate and momentum were constant at 1000, 0.5, and 0.1, respectively. The results show that the learning cycles were 49 epochs, and the best validation performance was found at 43 epochs with the MSE at 1027.99. Table 5 shows the outcome of the coefficient (R^2^) of the training and testing data phases. The neural network confirms the precise tools with perfect prediction accuracy in the responses of genotypes for coffee crop yield based on the measured indices. Thus, the ANN model was demonstrated to provide a perfect agreement between the exact and approximate coffee productivity. However, the prediction potential of productivity by ANN (0.9524) appeared to show higher accuracy than MLR (0.9235). MLR and ANN were compared through machine learning techniques using historical areas, productivity zones, rainfalls, relative humidity, and minimum and maximum temperature for the recent 180 months.

**Table 5:** Error and R^2^ (RSQ), RMSE (ton), and MSE (ton) in testing datasets of ANN model.

Model	R^2^	RMSE	MSE
ANN	0.9524	0.0784	1027.99

According to the study, MLR and ANN have focused on practical interpolation methods. RMSE and MSE cross-validation were accomplished to assess the efficiency of each month (168 months). It was also estimated through the average of RMSE and MSE. However, the cross-validation of MLR showed that the average values of RMSE and MSE are 0.0618 and 0.0038, respectively. Nonetheless, in comparison to the full model of coffee prediction for 180 months, RMSE was 0.0784, and MSE was 0.0061 tons, respectively. The results showed that both of them were not significantly different.

## Discussion and conclusion

The crop yield of cherry coffee varies because of several factors such as the cultivated area, amount of rainfall, temperature, and RH. These conditions affect the cherry coffee yield from each month's crop. Prediction is necessary to forecast the productivity accurately and to satisfy customer demand. Previously, farmers and entrepreneurs lack knowledge regarding the future cherry coffee yield, as well as which factors can change the cherry coffee crop yield each month. For this reason, production planning to match the supply to the demand is rather difficult. However, if farmers and entrepreneurs have sufficient data and can utilize it using machine learning models, they may realize more accurate estimate of the crop yield.

In this work, MLR and ANN were used to analyze and predict the coffee crop yield using data from 2004 to 2018, in which input data for 132 months was used for training, 24 months for validation, and 24 months for testing. Calculation of the cherry coffee crop yield required MATLAB programming. The MLR equation was derived from the full model consisting of six variables. The data shows that the area (X_1_) ranged between 42,216 and 20,158 acres with an average of 34,003 acres, playing an essential role in cherry coffee production. The available area is a crucial component for coffee tree planting because it determines the farmer’s cherry coffee productivity. The productivity zone (X_2_) shows data ranging between 37,710 and 14,513 acres, with an average of 23,921 acres. This is also essential because it indicates how much the farmer can earn from the cherry coffee yield^73^. The rainfall data (X_3_) is from 0 to 509.8, but the average rainfall is 1830 mm per month. The amount of RH (X_4_) is between 55.0 and 82.0%RH, and the mean is 76.0%RH which is suitable for coffee cultivation^74^. Moreover, the temperature (X_5_ and X_6_) is a factor that could affect coffee production and is shown in the data to be between 5.10 and 40.0 °C with an average of 25.5 °C^75^.

The correlation was checked between independent variables. As in Table 2, there are some high correlations, such as those between the minimum temperature and productivity, the area and productivity zone, and the productivity zone and productivity, with values of 0.9175, 0.8119, and 0.7775, respectively. For this reason, it was necessary to put all the variables into the model or may represent them with VIF < 10 values (variance inflation factor). The assumption of MLR was adjusted and estimated from $\widehat{Y}$' = 0.2850 + 0.0402X_1_ + 0.3128X_2_ − 0.0486X_3_ − 0.4040X_4_ − 0.6144X_5_ + 1.1065X_6_ with 0.9235 for the standard error of estimation. The R^2^ and Durbin‒Watson values confirmed the appropriateness of the MLR equation, as shown in Table 3. R^2^ and Durbin‒Watson values were 0.9235 and 1.56, respectively which are suitable for this MLR model. Meanwhile, R^2^ in Table 3 also indicates that the coffee variable substantially explains the changes in the crop yield, while the Durbin‒Watson test reveals a low autocorrelation for the predictive errors.

The importance of each input variable on the yield was also analyzed based on the MLR mathematical expression. The equation coefficients showed that Tmin monthly variable (X_6_) was the most important (1.1065). The second-most important factor was Tmax monthly (X_5_) with a coefficient of − 0.6144. The relative humidity (X_4_), the productivity zone (X_2_), and monthly total rainfall (X_3_) were inferior, with values of − 0.4040, 0.3128, and − 0.0486, respectively. Lastly, the area (X_1_) was shown to be the least essential variable (0.0402).

**Figure 6:**
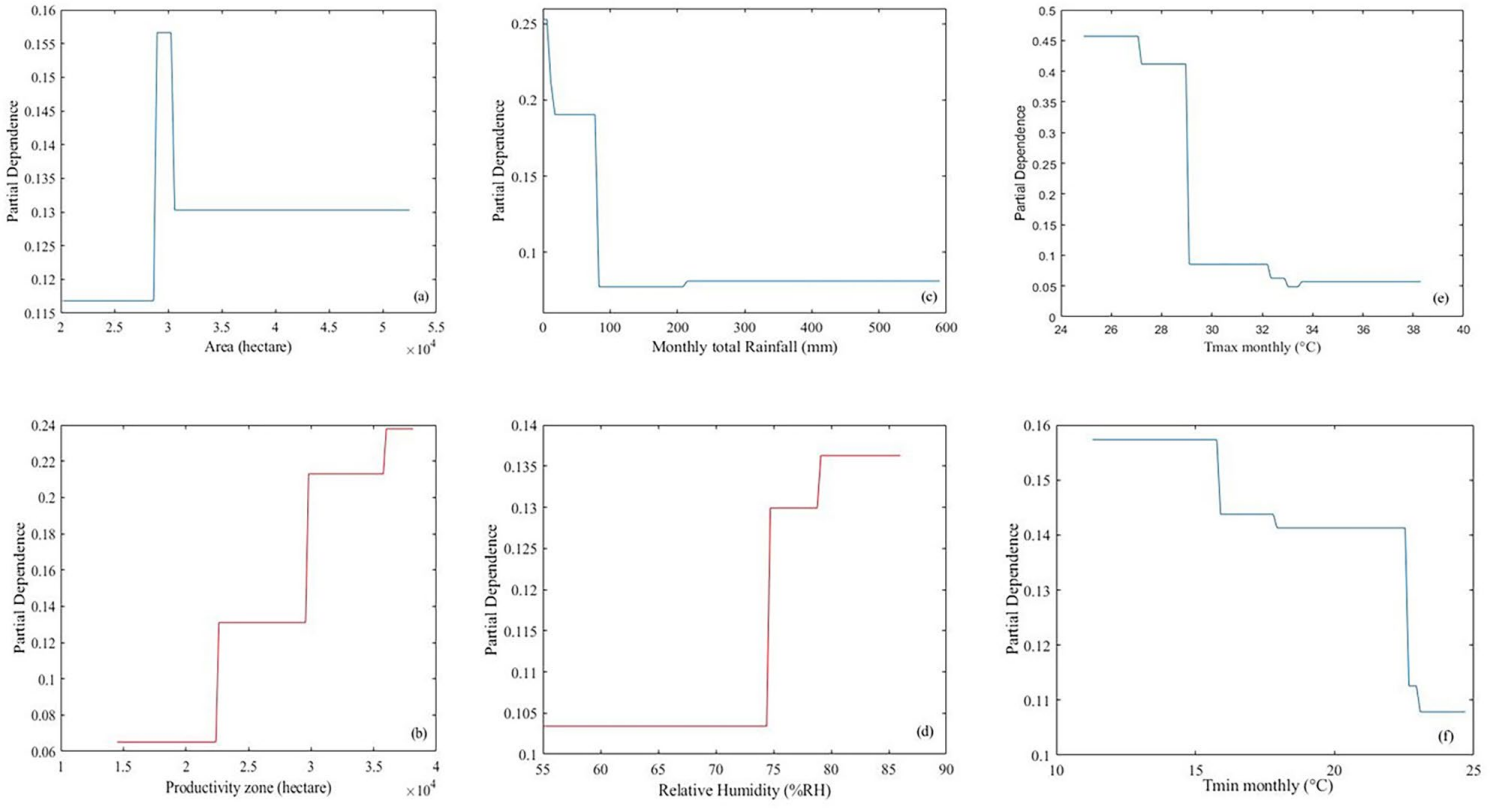
One-way PDPs of input variables on prediction of crop yields.

Similarly, for the ANN model, the relative importance of each input variable was evaluated based on one-way partial dependence plots (PDPs), as shown in Fig. 6. It can be seen that the monthly Tmax variable (X_5_) was the most important, in which the PDP value varied from 0.45 to 0.05. Similarly, the second most influential variable was monthly total rainfall (X_3_), with PDP values of 0.25 to 0.05. The third variable was relative humidity (X_4_) of 0.138 to 0.103. Additionally, the last three variable groups were productivity zone (X_2_), area (X_1_), and Tmin monthly (X_6_), showing the PDP values varied from 0.240 to 0.07, 0.158 to 0.118, and 0.160 to 0.110, respectively^76,77^. In Fig. 6e, when the Tmax monthly variable increased, cheery coffee's productivity was lowered, especially when it was higher than 29 °C. In other words, if the temperature was less than 29 °C, the productivity of cheery coffee was improved. From Fig. 6b, if we have more productivity zones, productivity will increase accordingly. From Fig. 6d, the productivity would be high if the cherry coffee experienced high relative humidity. On the contrary, if the amount of rain were too large, it would negatively affect productivity. Based on the PDPs in Fig. 6c, the cherry coffee should rain at less than 100 mm per month, leading to a high crop yield.

Likewise, the ANN method can obtain the input dataset from the available data. Then, the data was imported from the MATLAB workspace, and a variable was selected. A network or data was created to random the hidden layer until the performances of the various ANN configurations were known. The best performance is shown in Table 5, with values of 0.9524 and 0.0784 ton for R^2^ and RMSE, respectively. The results of both models indicate that the cherry coffee crop yield can be accurately predicted if the farmers or entrepreneurs are able to utilize these data.

In comparing MLR and ANN performances with other agricultural-related predictions, the R^2^ of the MLR was better than the ANN in some cases due to its limited number of input datasets. This was so for Bayat et al. work^78^ on predicting tree survival and mortality in the Hyrcanian forest. The six input variables were used for 9 years of prediction, including the wind velocity and direction, diameter at breast height, basal area of larger trees, diameter, topographic wetness index, and temperature. The R^2^ of ANN and MLR were 0.6790 and 0.9200, respectively. Ustaoglu et al.^79^ used MLR and ANN for temperature forecasts through daily mean, maximum, and minimum temperature in time series. The R^2^ of ANN was 0.8590, lower than that of MLR (0.9025).

The R^2^ of ANN was generally found to be better than that of MLR when many datasets were used, for example, in predicting rainfall^75,80^, and maize yield through the temperature and precipitation data^81^, monthly pan evaporation and soil temperatures^82,83^, the relative humidity of climate in Turkey^84^, and crop prediction^85,86^, as shown in Table 6. For the cherry coffee crop yield by MLR and ANN in this work, the R^2^ were 0.9235 and 0.9524, respectively, while RMSE was 0.0784 ton, showing the suitability for crop yield prediction.

**Table 6:** Comparison of MLR and ANN models for other agricultural production.

No	References	Subject	Number of data	Variables	R^2^		RMSE
					ANN	MLR	
1	Bayat et al.^78^	Predicting tree survival and mortality in the Hyrcanian forest of Iran	9 years	Wind velocity and direction, Diameter at breast height, Basal area of larger trees, Diameter, Topographic wetness index, Temperature	0.6790	0.9200	0.9850
2	Ustaoglu et al.^79^	Forecast of daily mean, maximum and minimum temperature time series	14 years	Temperature	0.8590	0.9025	0.9400
3	Ilaboya and Igbinedion^75^	Prediction of monthly maximum rainfall in Benin city, Nigeria	34 years	Rainfall	0.9999	0.1755	0.7800
4	El-Shafie1 et al.^80^	Rainfall forecasting	10 years	Rainfall	0.8110	0.4160	0.5168–0.6800
5	Matsumura et al.^81^	Maize yield forecasting	43 months	Temperature and precipitation data	0.8230–0.9540	0.7600–0.9350	0.8770
6	Patle et al.^82^	Monthly pan evaporation modelling	84 months	Temperature, Relative humidity, solar sunshine hours	0.8900	0.8800	0.3300
7	Kisi et al.^83^	Modeling soil temperatures at different depths	300 months	Temperature, Solar radiation, Wind speed, Relative humidity, and Soil temperature	0.9564–0.9940	0.8118–0.9761	–
8	Yasar et al.^84^	Estimation of relative humidity	144 months	Relative humidity	0.9624–0.9965	0.7379–0.9489	–
9	Li et al.^85^	Prediction of crop and soybean	1059 countries	Area	0.5329–0.9409	0.5041–0.9216	0.7501–0.9299
10	Han et al.^86^	Crop evapotranspiration prediction	24 months	Atmospheric pressure, Wind speed, Temperature, Relative humidity, Sunshine hours, and precipitation	0.8700	0.7900	0.9857

MLR and ANN were effective in forecasting the crop yield of cherry coffee. These methods can be utilized from upstream to downstream in coffee planning to respond to customer demand. Moreover, they can reduce the risk of insufficient production in the high season and improve future efficiency and effectiveness for industrial and agricultural products.

Concerning limitations, this research considered only six input datasets, which were the area, amount of rainfall, temperature, and RH that affected the efficiency of increased crop yield. More related data input such as farm location, irrigation water depth applied, precipitation, solar radiation, potential evapotranspiration, soil moisture, land cultivated, pH, nitrogen, phosphorus, potassium, air pressure, wind speed, and climate variables should also be considered. The crop yield prediction could be improved further^87–89^.

Once the crop yield and amount of cherry coffee are accurately predicted for future works, farmers or entrepreneurs can prepare their manufacturing resources for later stages of production planning accordingly. These may include (i) color sorters to classify the color shade of cherry coffee for higher added value and (ii) dryer machines to reduce the moisture content in the cherry coffee.

The ANN algorithm has been demonstrated to be effective in making predictions of production rates/yields from industries of coffee plantings. However, the dataset used may be imbalanced. It is essential to note that, in identifying the optimal granularity and refining the imbalanced dataset, combining granular computing and ML techniques is of great interest^90,91^. In general, the predictions of developed ML models are based on knowledge learning from the complex multi-dimensional relationships between features and target(s)^92^. When an imbalanced, small dataset is employed to train and develop an ML model, the developed model would have a poor ability to make predictions. An innovative approach of combining granular computing with ML or deep learning may be considered further, in which the dataset’s quality and efficiency could be improved via clustering examples in the best granularity, sampling, and refining into a more balanced example set, such as those deployed in services planning under social manufacturing context and bi-level model for risk classification of respiratory diseases^90–94^. Additionally, reinforcement learning techniques may be considered to predict agricultural production. The learning process happens along the way with six main components of (i) agent, (ii) action, (iii) environment, (iv) state, (v) policy, and (vi) reward. It was successful for order acceptance decision of mass-individualized printed circuit board manufacturing^95^. In this research, only six main input features were considered. Therefore, reinforcement learning with more complex datasets may be considered for cherry coffee and other agricultural industries in the near future.

## Materials and methods

### Research framework

**Figure 7:**
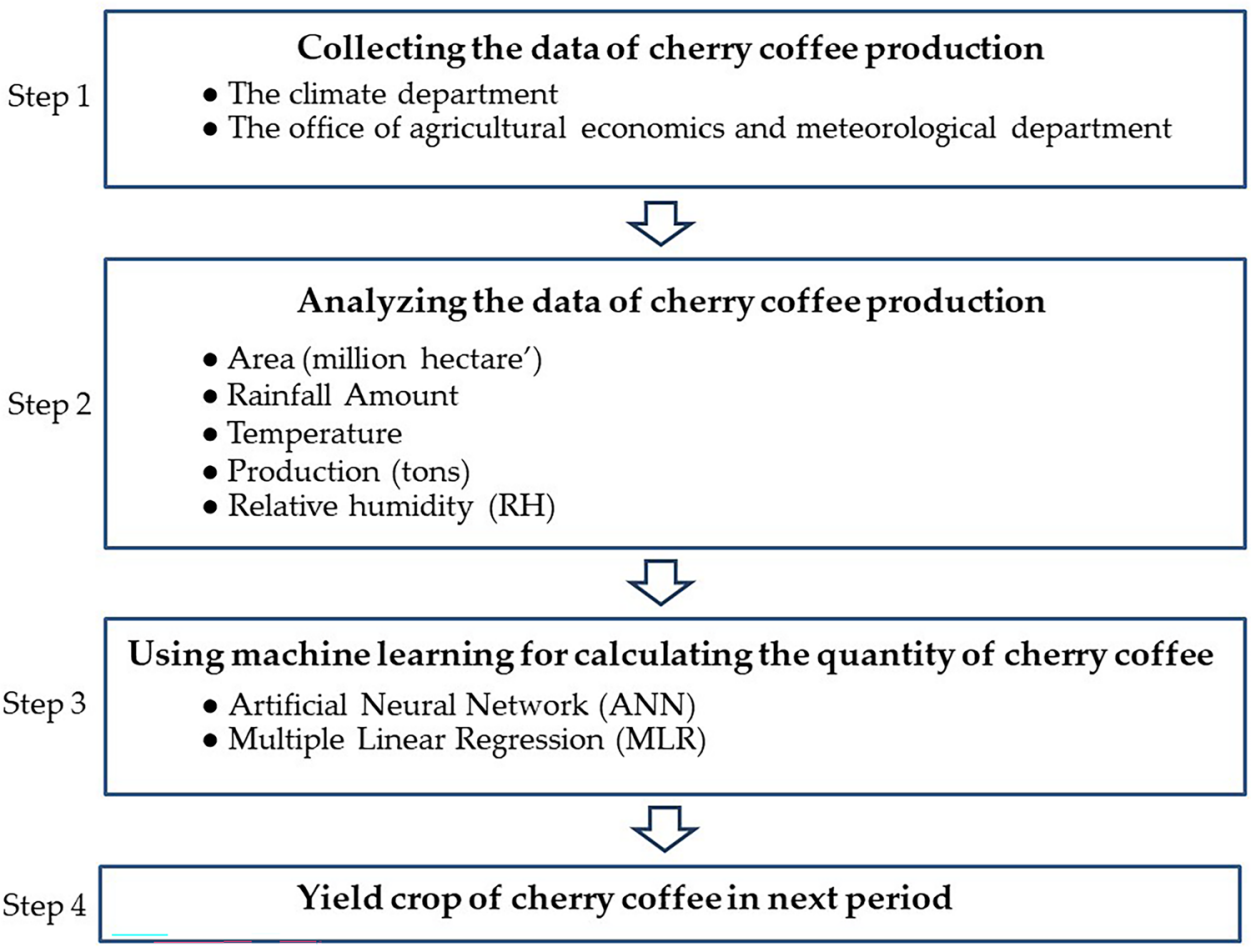
Research framework for cherry coffee prediction.

This study focuses on predicting cherry coffee. The scope of this study covers four steps from plantation until harvesting (see Fig. 7).

### Data collection and treatment

The data from the Climate Department included rainfall, RH, and minimum and maximum temperature. The Agricultural Economics Office and Meteorological Department provided the area and productivity zone data. The input dataset consisted of the area, rainfall, temperature, and (RH), while the output consisted of the crop yield of cherry coffee. The input and output data were normalized within the range 0‒1 using

1 $$N=\frac{\left(X-{X}_{min}\right)}{\left({X}_{max}-{X}_{min}\right)},$$

where *N* is the normalized data; *X* is the measured value: Xmin and Xmax are the minimum and maximum values. The ANN performance must be estimated by means of a validation dataset. The selected ANN was tested, and its performance was compared using the coefficient of determination (R^2^), the mean squared error (MSE), and the percentage error.

### Statistical and MLR model

MLR is one of the supervised learning groups that can generate the model for constructing a relationship between two or more interpretive variables (independent) and response variables (dependent) via appropriate linear equations into the observed data. The model for MLR is:

2 $${y}_{i}={b}_{0}+{b}_{1}{X}_{i,1}+{b}_{2}{X}_{i,2}+\cdots +{b}_{k}{X}_{i,k}+{e}_{i},$$

where Y_i_ is the dependent variable; b_0_ is a constant (intercept); X_i,k_ is an independent variable; b_k_ is the vector of regression coefficients (slope); and e_i_ is random measured errors.

### Data set, training and selection of optimal on figuration

The variables data were randomly split into three groups to prevent overfitting in the training step because a high number of neurons existed in the hidden layer. Training, validation, and testing of models were based on 70%, 15%, and 15% of the data, respectively^68^. In the meantime, during neural network training, the algorithm provided the minimum or maximum performance via the shortest path to yield the network's size. At the same time, backpropagation was performed through the training set in order to update the rule to seek and find the minimum mean square error over the training set.

The best point of generalization form validation was obtained from a training stop when the error started to increase until the final increase point. Next, the networks were trained with the testing set, which was also compared with the output dataset with the desired output, and the model weight was fixed^96^. After the training epoch, the mean square error of the network running of the validation set was calculated. Then, the neurons will apply a specific linear function in the hidden layer and a specific linear function in a hidden layer to collect the linear combination and bias. Finally, the output data will give the predicted model.

The validated model was evaluated based on the observed and predicted coffee crop yield difference. The 180 months of data on coffee production were randomly evaluated through a k-fold cross-validation technique but evenly partitioned into 168 months of the dataset. The dataset will be further randomly evaluated through three rounds of MLR and ANN (ten processing elements and two hidden layers) training. Each dataset was individually used as validation, while the enduring ones were treated as training datasets. From the k-fold cross-validation, RMSE and MSE were evaluated to determine the models’ performance:

3 $$\mathrm{RMSE}=\sqrt{\frac{1}{n}\sum_{i=1}^{n}[E\left({x}_{i}\right)-M\left({x}_{i}\right){]}^{2}},$$

4 $$\mathrm{MSE }=\frac{1}{ n}\sum_{i=1}^{n}{\left(Error\right)}^{2},$$

where *n* is the sample size of the testing dataset, while *E* (x_i_) and *M*(x_i_) are interpolated and observed values, respectively.

### Prediction of crop yield

In this model, the crop yield was considered to assess indices through the input and output data. A partial dependence plot (PDP) was performed to evaluate each variable as an independent variable during the neural network process^76,77^. The relative importance of the parameters for each index was computed by dividing the value of its importance by the highest importance value. Consequently, the model with the largest R^2^ and the smallest RMSE was considered the best.

The ratios of the correlation between standard errors and mean values were calculated by evaluating the accuracy in terms of repeatability and similarity for yield-based with the maximal indices up to 1000 bootstrap samples similar to the coefficient of variation (CV). A low CV indicates a selection index that is more accurate^65^. All implementations were accomplished using the neural network toolbox in the MATLAB package. Therefore, in the present study, MATLAB version R2019b, with Windows 10 and a 64-bit machine, was used to run the MLR and ANN models.

## Data Availability

The datasets used and/or analyzed during the current study available from the corresponding author on reasonable request.
